# Diverse Underlying Mechanisms and Sex Differences Found in Translational Models of Cannabinoids Use: Towards Validation in Human Studies

**DOI:** 10.3390/ijms242316586

**Published:** 2023-11-22

**Authors:** Sari Goldstein Ferber, Aron Weller

**Affiliations:** 1 Psychology Department and the Gonda Brain Research Center, Bar-Ilan University, Ramat Gan 5290002, Israel; aron.weller@biu.ac.il; 2 Department of Psychology and Brain Sciences, University of Delaware, Newark, DE 19716, USA

This Special Issue represents a continuation of our previous Special Issue entitled “Endocannabinoids, Cannabinoids and Psychiatry: Biological Mechanisms”.

As stated in the summary of this Special Issue: “During the past decade, scientific knowledge on the role of endocannabinoids and cannabinoids in the optimal functioning of the human brain has advanced considerably. Based on recent scientific findings, the endocannabinoid system (ECS) is regarded as a modulatory system of brain connectivity [1] and top-down–bottom-up synaptic pathways’ protection against extreme inhibitory or excitatory cortical and sub-cortical conditions. When dysregulation of the ECS is apparent, such extreme conditions have been suggested to underlie psychopathology [2,3]. Additionally, various researchers, including those who have used animal models, e.g., [4,5,6], have recently demonstrated the stabilizing effects of cannabinoids and endocannabinoids on mood disorders, anxiety, depression and schizophrenia, social phobia, personality disorders, bipolar disorder, and post-traumatic stress disorder. Research on the role of endocannabinoids in the developmental course, and remission of psychiatric disorders has also been recently advanced”.

In this Editorial we discuss four empirical studies and three review papers on cannabinoids and their impacts on psychiatric disorders, included in this Special Issue. All the empirical studies presented used animal models and two of them reported sex differences in the effects of cannabinoid-based treatments.

Vitale et al. (contribution 1) reviewed current knowledge on the complex pharmacological profile of cannabidiol (CBD) in the context of its therapeutic effects on several neurological and neuropsychiatric disorders. Their review discusses many molecular targets associated with these disorders, such as the Cys-Loop Superfamily of Ligand-Gated Ion Channels, TRP Channels, GPCRs, s1R, and PPARg, modified by CBD. After presenting data on CBD’s effects on epilepsy, Alzheimer’s disease, Parkinson’s disease, depression, anxiety, drug addiction, autism spectrum disorder, and psychotic disorders, the authors describe, in chemical details, several potential candidate mechanisms for pharmacological treatment. Based on their results, CBD is described as “a multitarget ligand with an additive and consistent pharmacological profile (polypharmacology)”. 

In an animal model study of anxiety-like behavior, Papagianni et al. (contribution 2) studied the effects of CBD on the reappearance of fear after extinction appeared to succeed and on resistance to extinction following stress. Specifically, they determined the effects of CBD on delayed extinction and on the later reappearance of fear, and on resistance to extinction resulting from immediate extinction. CBD prevented the unprompted reappearance of fear after delayed extinction and corrected the problems caused by immediate extinction. The underlying pharmacological mechanisms are unknown; nevertheless, these findings support the potential role of CBD as an anxiolytic agent. 

Using a Wistar Kyoto (WKY) rat model of depression, Hen-Shoval et al. (contribution 3) explored the behavioral effects of a forced swimming test (FST) and the possible underlying mechanisms of action of the synthetic analogue of cannabidiolic acid: CBDA-ME. This study expanded upon work previously reported by this group in male WKY rats [7], extending to female WKY rats the finding that the oral administration of CBDA-ME produces antidepressant-like effects, albeit at higher doses than in males. A CB2 receptor antagonist prevented the antidepressant-like effect in females, while it was not effective in males. In females, CBDA-ME also increased blood levels of BDNF and a few endocannabinoids, and decreased FAAH expression in the hippocampus. The authors proposed a possible cannabinoid-receptor-mediated biological mechanism for the acute antidepressant-like effect in females and noted that the results support the potential therapeutic value of this cannabinoid. 

Using rats, Portugalov et al. (contribution 4) examined the potential mechanisms underlying the effect of early life stress (ELS) on future depression. They measured the expression of microRNAs (miRs) associated with depression in the medial prefrontal cortex (mPFC), hippocampus (CA1), lateral habenula, and dorsal raphe, as well as the expression of serotonergic (htr1a and slc6a4) and endocannabinoid (cnr1, cnr2, and FAAH) genes in the mPFC. They demonstrated that that the FAAH inhibitor URB597 reversed depressive-like behavior, as well as reversing ELS-induced decreases in mPFC miR-135a in females and miR-16 in males. In addition, URB597 treatment in ELS females corrected decreased FAAH mRNAs and increased cnr2 in the mPFC. Thus, novel candidate mechanisms were reported, and the results were sex-dependent. 

The results of these studies warrant validation in humans, for which consistent results may have importance for the treatment of depression, especially in women, a subpopulation with a high prevalence of depressive disorders [8,9].

In another animal model study, Charalambous et al. (contribution 5) showed that the hormone ghrelin and its growth hormone, secretagogue receptor (GHSR1A), mediate some of the rewarding/reinforcing effects of cannabinoids. These findings follow this research team’s previous reports on interactions between ghrelin/GHS-R1A and endocannabinoids (arachidonoyl ethanolamide/anandamide/AEA and 2-arachidonoylglycerol/2-AG), along with dopamine, opioid, and γ-aminobutyric acid/GABA systems within the rat mesolimbic system [10,11,12,13,14,15]. This study shows potential mechanisms underlying the crucial role of the endocannabinoid system in depressive conditions, which are known to include anhedonia and reduced reward-seeking tendencies [16,17,18].

Bright and Akirav (contribution 6) reviewed the literature on the neuromodulation of different parts of the ECS (endocannabinoid ligands, degrading enzymes, and receptors) in relation to specific depression-like behaviors in rodent models and in people with depression. The literature reviewed included studies on the effects of the agonists and antagonists of cannabinoid receptors and on the effects of CBD, studies on the modification of parts of the ECS in depressed patients (endogenous ligands and cannabinoid receptors), and studies on genetic ECS variants that are known to be related to depression. The studies in rodents showed that “both direct and indirect activation of ECS components and CB1r in particular have an antidepressant potential, whereas deficits in ECS signaling may have depressive effects”, while the results from studies in humans were inconclusive. 

In a review paper on the effects of cannabinoids on the neuroinflammatory component of depression, Zádor et al. (contribution 7) present data on natural and synthetic cannabinoids that increase pro-inflammatory cytokines and their association with the kynurenine pathway (KP). The pre-clinical and clinical evidence reviewed support the upregulation of pro-inflammatory cytokines, which may then over-activate the KP. This could play a role in the decreased serotoninergic function often found in conditions diagnosed as major depression in humans. The authors therefore suggest that the KP may be “the missing link between cannabinoid-induced inflammation and depressive symptoms”. 

In conclusion, research using animal models supports the potential therapeutic effects of cannabinoids and the curative potential of the ECS in neuropsychiatric disorders. Many biological mechanisms are involved, allowing for the application of diverse novel pharmacological and pharmacogenetic approaches. The importance of a gender-medicine approach emerges from the literature presented herein. The translational results included in this Special Issue may serve as a basis for validation studies in human clinical trials.
